# Cofilin-1 levels and intracellular localization are associated with melanoma prognosis in a cohort of patients

**DOI:** 10.18632/oncotarget.25303

**Published:** 2018-05-08

**Authors:** Candelaria Bracalente, Adriana R. Rinflerch, Irene L. Ibañez, Francisco M. García, Victoria Volonteri, Gastón N. Galimberti, Fabio Klamt, Hebe Durán

**Affiliations:** ^1^ Gerencia de Investigación y Aplicaciones, Comisión Nacional de Energía Atómica, (B1650KNA) San Martín, Buenos Aires, Argentina; ^2^ Consejo Nacional de Investigaciones Científicas y Técnicas, (C1425FQB) CABA, Buenos Aires, Argentina; ^3^ Dermatología Experimental, Servicio de Dermatología, Hospital Italiano de Buenos Aires, (C1199ABB) CABA, Buenos Aires, Argentina; ^4^ Escuela de Ciencia y Tecnología, Universidad Nacional de San Martín, Campus Miguelete, (B1650HMP) San Martín, Buenos Aires, Argentina; ^5^ Servicio de Anatomía Patológica, Hospital Italiano de Buenos Aires, (C1199ABB) CABA, Buenos Aires, Argentina; ^6^ Laboratório de Bioquímica Celular, Departamento de Bioquímica, Instituto de Ciências Básicas da Saúde, Universidade Federal do Rio Grande do Sul, (90035 003), Porto Alegre, Brazil

**Keywords:** cofilin-1, melanoma, metastasis, nuclear localization, prognosis

## Abstract

Melanoma is an aggressive cancer with highly metastatic ability. We propose cofilin-1, a key protein in the regulation of actin dynamics and migration, as a prognostic marker. We determined cofilin-1 levels in a retrospective cohort of patients with melanomas and benign lesions of melanocytes (nevi) by immunohistochemistry. Higher cofilin-1 levels were found in malignant melanoma (MM) with Breslow Index (BI)>2 vs MM with BI<2, melanoma *in situ* (MIS) and nevi and also in MM with metastasis vs MM without detected metastasis. Kaplan-Meier survival curves were performed, clustering patients according to either the type of melanocytic lesions or cofilin-1 level. Survival curves demonstrated worse prognosis of patients with high vs low cofilin-1 levels. TCGA database analysis of melanoma also showed low survival in patients with upregulated cofilin-1 mRNA vs patients without alteration in CFL1 mRNA expression. As cofilin-1 has a dual function depending on its intracellular localization, we evaluated nuclear and cytoplasmic levels of cofilin-1 in melanoma and nevi samples by immunofluorescence. MM with high Breslow index and metastatic cells not only presented cytoplasmic cofilin-1, but also showed this protein at the nucleus. An increase in nuclear/cytoplasmic cofilin-1 mean fluorescence ratio was observed in MM with BI>2 vs MM with BI<2, MIS and nevi.

In conclusion, an association of cofilin-1 levels with malignant features and an inverse correlation with survival were demonstrated. Moreover, this study suggests that not only the higher levels of cofilin-1, but also its nuclear localization can be proposed as marker of worse outcome of patients with melanoma.

## INTRODUCTION

Malignant melanoma (MM) is one of the most aggressive cancers, with high metastatic ability and increasing mortality rates. Although melanoma accounts for less than 1% of skin cancer, it is responsible for almost 90% of skin cancer deaths. It is estimated that 91,270 new cases of invasive melanoma will be diagnosed in the U.S. in 2018 [1]. The staging system most often used for melanoma is the American Joint Commission on Cancer (AJCC) TNM system [2]. Early stage melanoma can be cured by surgery alone, but late stage metastatic disease has limited treatment options. Although new approaches that include immunotherapy and targeted therapies have led to improvements in patient outcomes [3–6], the advanced disease has still extremely poor outcomes with 5 year overall survival of less than 10% [7]. There is a highly significant decline in survival rates as melanoma thickness (also called Breslow index, BI) increase. Besides, the chances of relapse do not differ much between stage T2 and stage T3 patients; in addition, for very early stage melanoma (≤1 mm, T1a or T1b) the risk of recurrence ranges between 1% and 12% [8].

Conventional tissue biomarkers, such as BI, ulceration, mitotic rate and lymph node positivity, remain the backbone prognostic indicators in melanoma [9]. However, since recurrence rate is largely independent from stages defined by morphological and morphometric criteria, there is a strong need for identification of additional robust prognostic factors to support decision-making processes [8]. In this context, several immunohistochemical (IHC) markers are used in the clinical setting for the evaluation of melanocytic lesions (e.g. S100, HMB45, Melan-A/MART1 and MITF). While these markers are useful in differentiating melanocytic from nonmelanocytic neoplasms, they are not capable of reliably distinguishing neither benign from malignant melanocytic neoplasms nor primary from advanced/metastatic melanoma [10]. In addition, BRAF mutations occur commonly in both nevi and melanomas [11]. Thus, additional molecular markers of these cutaneous lesions are needed in order to accurately predict the behavior of melanoma in individual patients to monitor disease progression, recurrence, and response to treatment [12–14].

The ability of cells to migrate and invade the dermis is critical in metastatic melanoma and therefore an indicator of poor prognosis. Thus, proteins involved in transforming melanoma cells into a migratory phenotype can be proposed as prognostic markers. In this sense, high levels of cofilin-1, which favors migration by inducing cycles of actin polymerization/depolymerization [15], has been found in metastatic cells [16, 17]. In addition, cofilin-1 as an effector of transforming growth factor-β is an important contributor of epithelial mesenchymal transition programming, which confers invasive and metastatic properties during cancer progression [18]. In this regard, cofilin-1 overexpression was associated with increased migration in colon adenocarcinoma cells [19] and has been used as poor prognosis marker, related to metastasis, in non-small cell lung cancer patients [20, 21] and invasive breast cancer cells [17]. Besides, a previous work from our laboratory demonstrated in a melanoma model with different degree of malignancy a correlation among the high levels of cofilin-1 and the migratory, invasive and metastatic ability of cells [22], suggesting its relevance in melanoma progression.

The activities of different proteins can be controlled by their concentration and subcellular localization. Cofilin-1 is enriched in cytoplasmic locations as regulator of actin-cytoskeleton and cell locomotion [23]. However, cofilin-1 also has a bipartite nuclear localization signal (NLS) [24], which allows its involvement in transcriptional elongation and nuclear morphology and function, including chromosome regulation. Cofilin-1 also translocates actin, which lacks of a NLS, into the nucleus, where actin regulates transcription and gene activation [25]. However, the nuclear localization of cofilin-1 was not described in melanoma progression.

These evidences lead us to determine the levels and subcellular localization of cofilin-1 in melanoma human samples with different degrees of malignancy, in order to define this protein as predictor of advanced melanoma. In this sense, we demonstrated increased cofilin-1 levels associated with malignant features of melanoma, such as BI and metastasis. We also found an inverse correlation between cofilin-1 levels and patient survival. Remarkably, cofilin-1 was also observed in the nucleus of more malignant melanomas. These results allow us to propose cofilin-1 as a prognostic marker of melanoma.

## RESULTS

### Cofilin-1 protein levels and patients overall survival

The clinicopathological features of patients and cofilin-1 immunocontent, expressed as percentage of samples with low or high cofilin-1 for each condition, are summarized in Table 1. Results show a significant association between high levels of cofilin-1 and MM advanced stages (p<0.001), metastasis (p<0.01), increased number of mitosis per field (p<0.05) and BI (p<0.05).

**Table 1:** Epidemiological and clinical features of the cohort of patients with benign and malignant melanocytic lesions according to cofilin-1 immunocontent

Characteristics	Cofilin-1 Immunocontent	
	Low (< 0.08 A.U.)	High (> 0.08 A.U.)
**Cohort, n=42**	57.1 %	42.9 %
**Age, years**	58.6 ± 21.3	69.6 ± 16.6
**Sex**		
Men, n=22	59.1 %	40.9 %
Women, n=18	50 %	50 %
Undetermined, n=2	100 %	0 %
**Histological Type**		
Nevus, n=9	100 %	0 %
MIS, n=8	87.5 %	12.5 %
MM		
SSM, n=5	80 %	20 %
LMM, n=1	0 %	100 %
NMM, n=10	30 %	70 %
Undetermined MM, n=9	11.1 %	88.9 %
**Tumor Stage (TNM)**		
0, n=8	87.5 %	12.5 %
IA, n=4	50 %	50 %
IB, n=4	50 %	50 %
IIB, n=1	100 %	0 %
III, n=2	50 %	50 %
IV, n=9	22.2 %	77.8 %
Undetermined, n=5	0 %	100 %
**Metastasis**		
No, n=17	70.6 %	29.4 %
Yes, n=11	18.2 %	81.8 %
**Mitosis/Field**		
< 1, n=10	60 %	40 %
1-5, n=13	15.4 %	84.6 %
> 5, n=1	0 %	100 %
**Breslow Index**		
< 2 mm, n=10	50 %	50 %
> 2 mm, n=15	20 %	80 %

Metastasis includes lymph nodes and distant metastases. Abbreviations: LMM, Lentigo maligna melanoma; MIS, Melanoma in situ; MM, Malignant melanoma; SSM, Superficial spreading melanoma; NMM, Nodular malignant melanoma.

**Figure 1:**
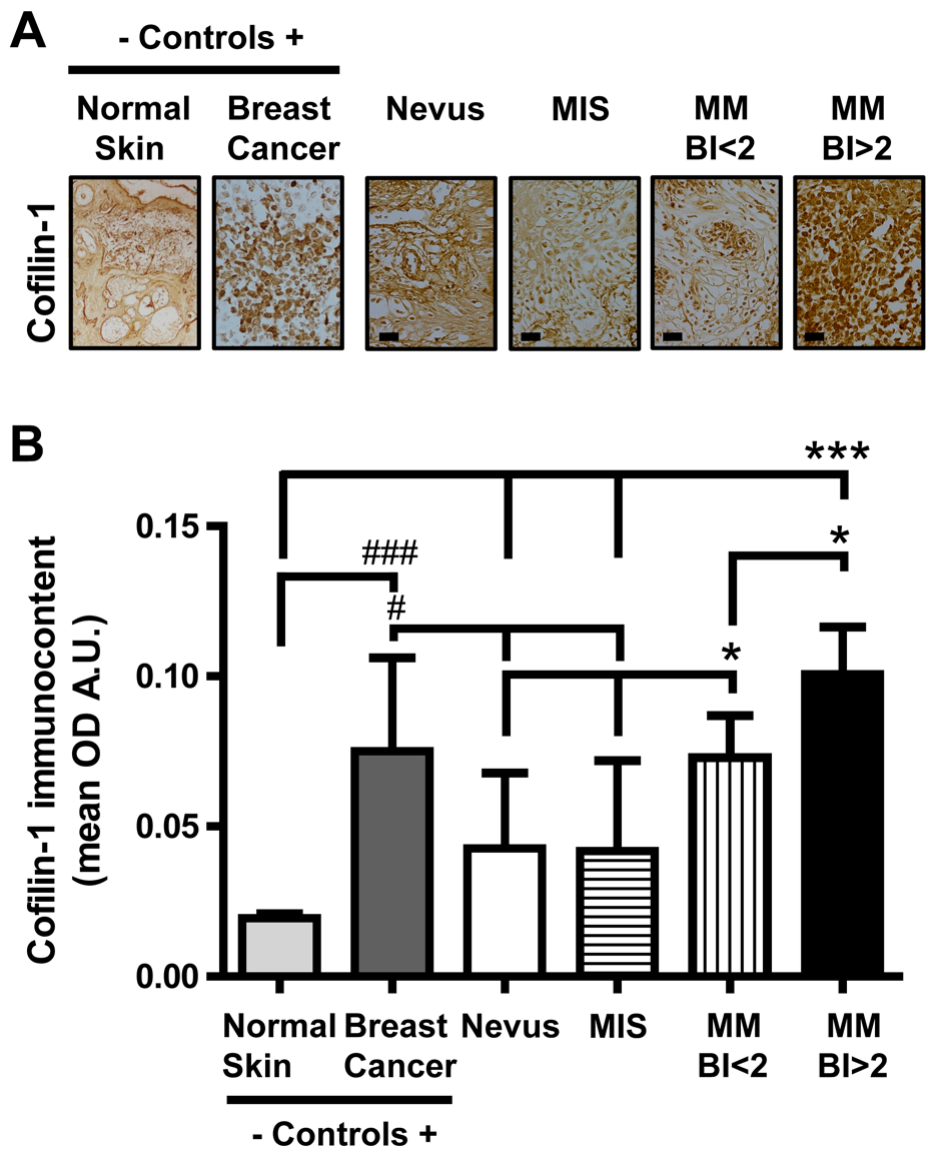
Cofilin-1 immmunocontent in benign and malignant melanocytic lesions **(A)** Representative IHC images in tissue sections of benign and malignant melanocytic lesions (microphotograph obtained with a 1000X magnification; bar represents 50 μm), normal skin (negative control, microphotograph obtained with a 40X magnification) and experimental breast cancer (positive control, microphotograph obtained with a 400X magnification). **(B)** O.D. mean values expressed as arbitrary units (A.U.) of cofilin-1 immunocontent in tissue sections. Data are expressed as mean ± SD. ^*^p < 0.05 MM BI<2 vs nevus and MIS; ^*^p < 0.05 MM BI>2 vs MM BI<2; p < 0.001 MM BI<2 vs normal skin; ^***^p < 0.001 MM BI>2 vs normal skin, nevus and MIS; ^###^p<0.001 breast cancer vs normal skin; ^#^p<0.05 breast cancer vs nevus and MIS.

Increased cofilin-1 immunocontent was detected by IHC in MM vs MIS and nevi (Figure 1A). The quantification of cofilin-1 immunocontent as O.D. mean values confirmed its significant increase in cofilin-1 for MM vs nevi and MIS, and for MM BI>2 vs all types of lesions (Figure 1B). The O.D. values of cofilin-1 per sample were distributed within a range of variation, between 0.009, the lowest value attributed to a nevus, and 0.13, the highest value obtained from a MM BI>2.

**Figure 2:**
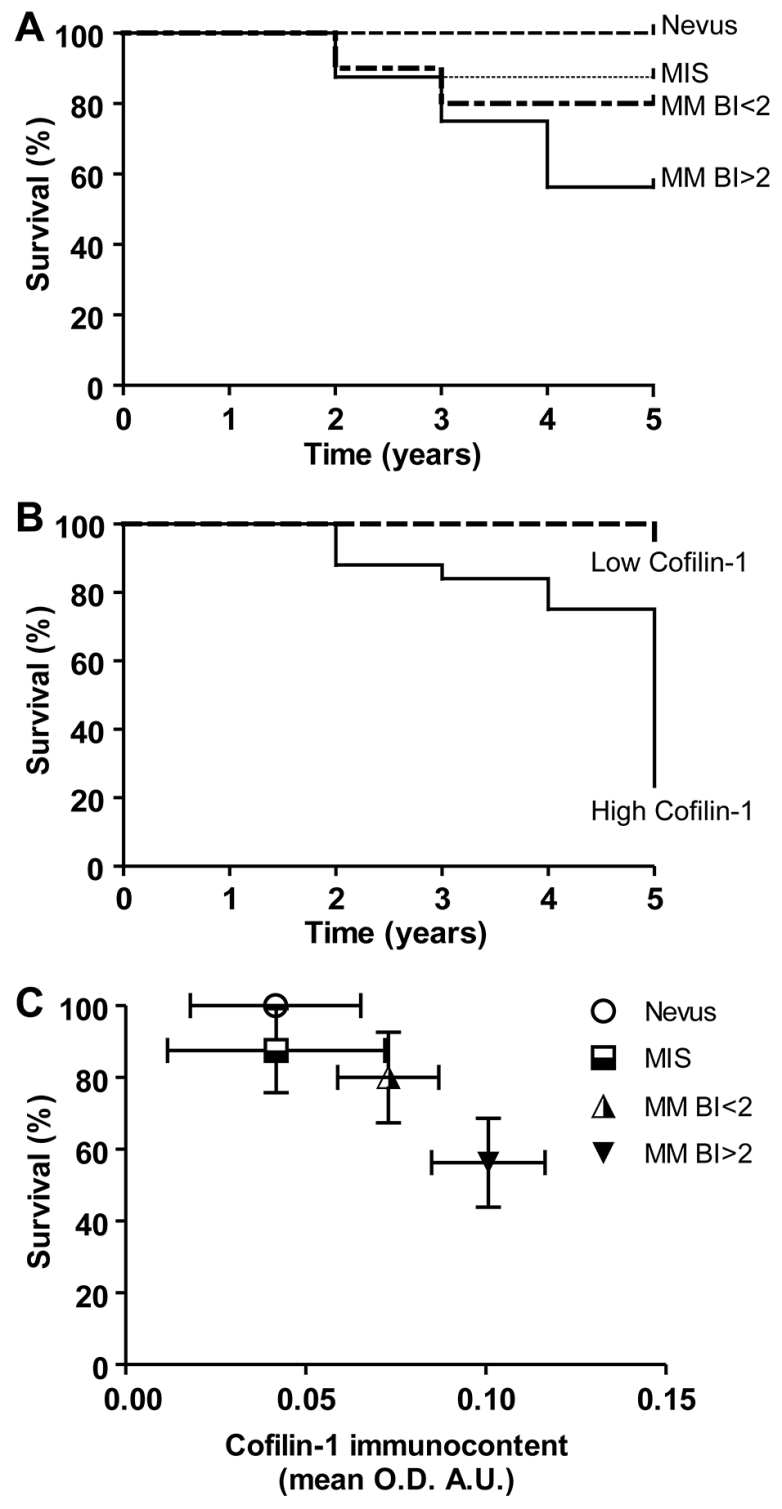
Kaplan–Meier survival curves of patients clustered according to the type of melanocytic lesions and to the cofilin-1 immunocontent **(A)** Overall survival in 5 years of patients clustered according to the type of lesions: nevus, MIS, MM BI<2 and MM BI>2. **(B)** In order to evaluate the cofilin-1 performance to discriminate between good and bad prognosis, patients were clustered according cofilin-1 immunocontent in low and high cofilin-1 with a cutoff of 0.08 A.U. of O.D. mean values and Kaplan-Meier survival curves were performed. **(C)** Correlation between survival percentage and cofilin-1 immunocontent. Data represent mean ± SD of survival percentage and cofilin-1 immunocontent. A significant correlation between both parameters resulted from the Spearman’s regression analysis (R=-0.73, p=0).

Kaplan-Meier survival curves of patients clustered according to the type of lesions: nevus, MIS, MM BI<2 and MM BI>2, and to the cofilin-1 immunocontent were performed. Survival analysis of patients with these different types of melanocytic lesions revealed significant differences (p < 0.05) among curves evaluated by log-rank test for trend (Figure 2A). Regarding cofilin-1 immunocontent, survival curves indicated that those patients who have higher cofilin-1 levels present lower survival rate at 5 years (p<0.0001) analyzed by both log-rank test and Gehan-Breslow-Wilcoxon test (Figure 2B). Moreover, a significant negative correlation between survival percentage and cofilin-1 immunocontent (Spearman Correlation: R=-0.73, p=0) was found (Figure 2C). Therefore, the levels of cofilin-1 can be associated with the melanoma outcome in this patient cohort.

### Cofilin-1 mRNA expression and patients overall survival from TCGA melanoma data set

**Figure 3:**
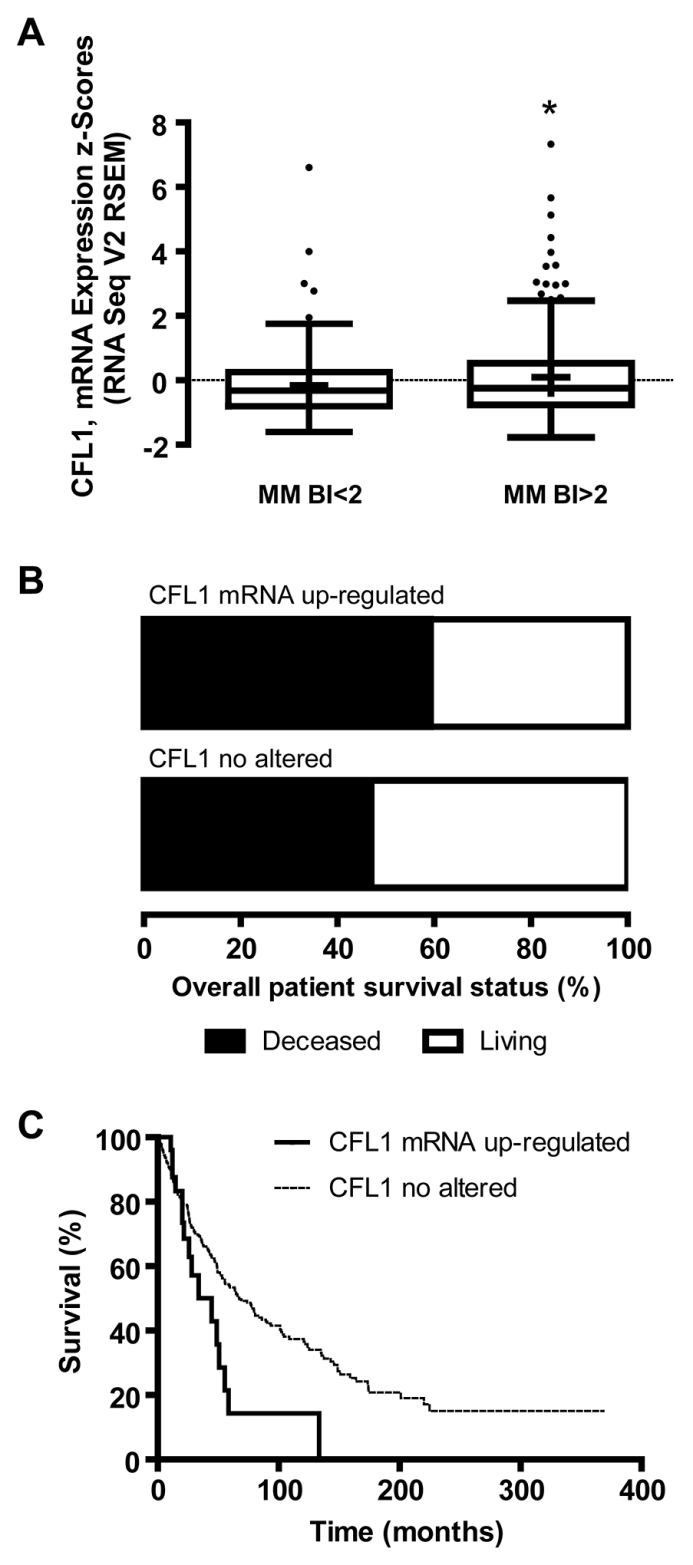
Cofilin-1 mRNA (CFL1) expression, BI and overall patient survival analyzed from TCGA melanoma data set **(A)** Tukey boxplot of CFL1 mRNA expression z-scores (RNA Seq V2 RSEM) vs BI clustered as MM BI<2 and MM BI>2. Mean is show as +. ^*^p < 0.05 MM BI>2 vs MM BI<2. **(B)** Overall survival status of patients with melanomas where CFL1 mRNA was up-regulated or no altered. **(C)** Kaplan–Meier survival curves of patients with melanomas where CFL1 mRNA was up-regulated or no altered.

Regarding the association of cofilin-1 levels and BI, a significant increased expression of CFL1 mRNA was observed in MM BI>2 compared with MM BI<2 (p<0.05) in the melanoma cohort from the TCGA Research Network (Figure 3A). Overall patient survival status analysis showed 59.3 % of deceased patients with melanomas where CFL1 expression was up-regulated vs 47 % of deceased patients with melanomas where CFL1 was no altered (Figure 3B). Kaplan-Meier survival curves analysis indicated that those who have higher CFL1 mRNA expression levels present lower survival rate (p<0.001) analyzed by both log-rank test and Gehan-Breslow-Wilcoxon test (Figure 3C). Remarkably, these results support those obtained in our cohort of patients, suggesting that not only at protein level but also at mRNA expression level, cofilin-1 can be associated with a worse prognosis in melanoma.

### Subcellular localization of cofilin-1 in malignant vs benign melanocytic lesions

**Figure 4:**
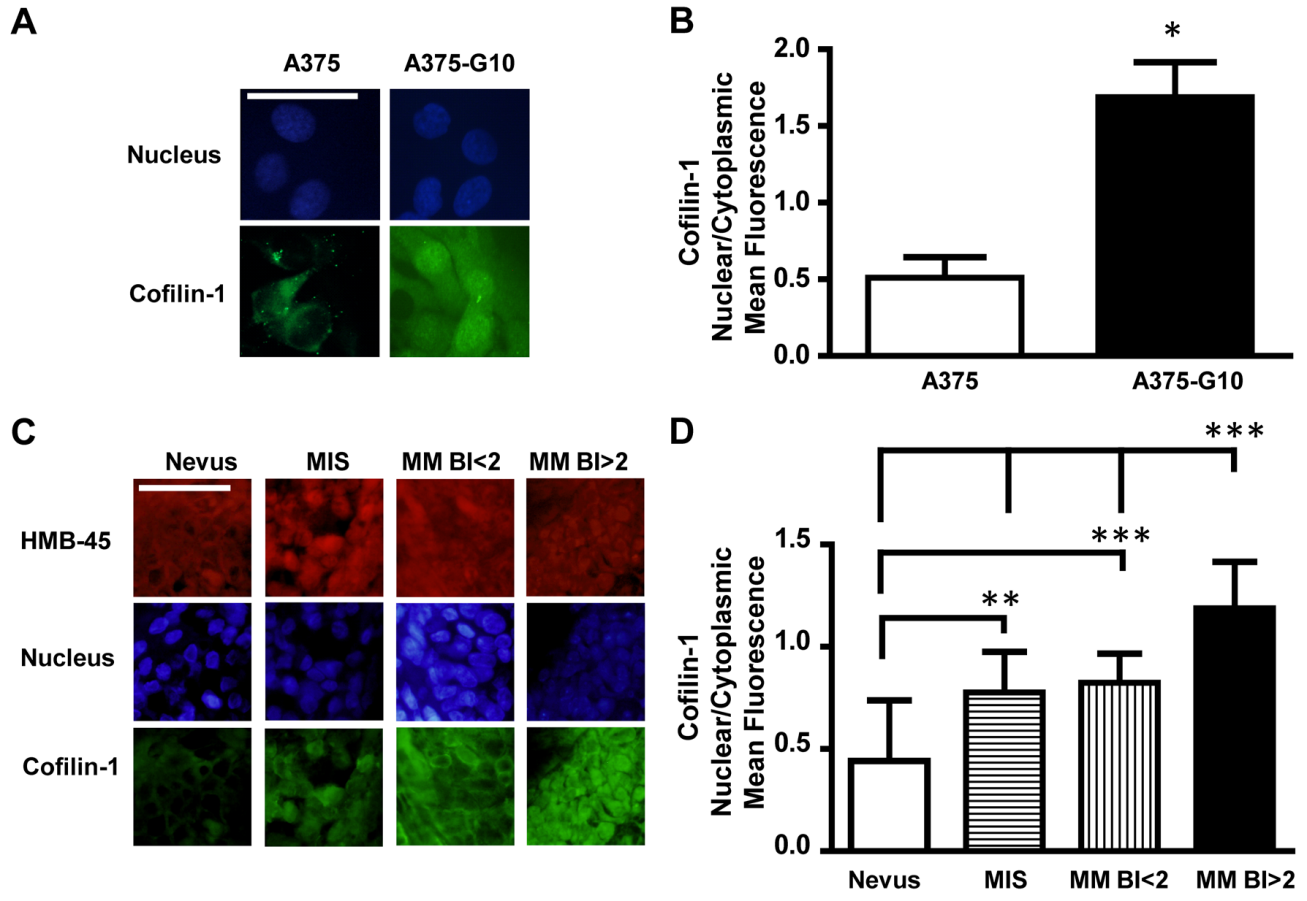
Cofilin-1 showed both cytoplasmic and nuclear localization in more aggressive melanomas **(A)** Representative immunocytofluorescence images of cofilin-1 with their corresponding nuclear staining using DAPI detection in non-metastatic A375 vs metastatic A375-G10 human melanoma cells. Bar represents 50 μm. **(B)** Quantification of cofilin-1 cytoplasmic and nuclear expression in A375 and A375-G10 cells showed as the ratio of nuclear/cytoplasmic mean fluorescence values. Data represent mean ± SD. ^*^p < 0.05 A375-G10 vs A375. **(C)** Representative images of cofilin-1 and HMB-45 (as a marker of melanocyte lineage), detected by immunohistofluorescence in tissue sections of benign and malignant melanocytic lesions of patients with their nuclear staining using DAPI detection. Bar represents 50 μm. **(D)** Quantification of cofilin-1 cytoplasmic and nuclear expression in tissue sections of benign and malignant melanocytic lesions showed as the ratio of nuclear/cytoplasmic mean fluorescence values. Data represent mean ± SD. ^**^p < 0.01 MIS vs nevus; ^***^p < 0.001 MM BI<2 vs nevus; ^***^p < 0.001 MM BI>2 vs MM BI<2, MIS and nevus.

Melanoma cells with different degree of malignancy exhibited differential subcellular localization of cofilin-1 detected by immunocytofluorescence (Figure 4A). Cofilin-1 was found localized both in cytoplasm and nucleus in metastatic A375-G10 cells, while it was mainly observed in cytoplasm in non-metastatic A375 cells. A significant difference on the ratio of nuclear/cytoplasmic cofilin-1 mean fluorescence was found between these metastatic and non-metastatic melanoma cells (Figure 4B).

Regarding cofilin-1 detection by immunohisto-fluorescence in benign and malignant melanocytic lesions, representative images of the immunofluorescence nuclear/cytoplasm values of each type of lesion, quantified as described in Materials and Methods section, are shown in Figure 4C. The ratio of nuclear/cytoplasmic cofilin-1 mean fluorescence exhibited significant differences between MM and MIS vs nevi. MM with BI>2 showed the higher ratio as compared with MIS and MM BI<2 (Figure 4D). Thus, more aggressive melanomas also exhibited increased nuclear levels of cofilin-1.

## DISCUSSION

Proteins involved in the induction of cell migration and invasion may be relevant as potential molecular markers of aggressiveness and bad prognosis. Particularly, we focused on cofilin-1, which is a key mediator of actin cytoskeleton polymerization and migration [26]. This is the first study in a cohort of patients with melanoma that correlates cofilin-1 levels with malignant features and survival. Molecular biomarkers are needed to optimize melanoma treatment due to their potential role in early diagnosis, prognosis and predictive response to therapies. The currently available melanoma biomarkers are mainly based on histopathological parameters. Breslow thickness has been demonstrated as one of the main clinicopathological parameters for predicting the outcome of patients with melanoma local lesions, which concomitant with mitotic index and lymph nodes allows defining melanoma staging [27]. However, there remains variability in prognosis among melanomas classified in the same stage, for example for stage IIA melanomas, the risk of relapse and mortality may approach 25%. This current criteria lack the ability to identify the individuals who will progress in each staging group [28, 29]. Therefore, during the last years the emphasis has turned to the study of molecular markers to achieve a better interpretation of endogenous mechanisms of melanoma and a more accurately prognosis, which could have implications in personalized therapies [30].

In this sense, we demonstrated an association between cofilin-1 levels and malignancy, measured by Breslow thickness, melanoma staging, mitotic index and presence of metastasis, in melanoma patients. This is in agreement with an *in vitro* previous study, where we reported high levels of cofilin-1 in migrating cells with metastatic ability compared with non-migratory neither metastatic control cells in an experimental model of human melanoma [22]. Besides, proteomic studies showed differential expression of cofilin-1 isoforms in two melanoma cell lines compared with a melanocyte cell line [31] and increased expression of cofilin-1 in metastatic lymph node compared with matched human primary cutaneous melanoma tissue of the same patients [32]. Furthermore, the analysis of cofilin-1 mRNA levels in the melanoma cohort from the TCGA Research Network supported the results obtained in our cohort of patients.

Moreover, we demonstrated an inverse correlation between cofilin-1 and survival, both at protein and mRNA expression levels in melanoma. The association of cofilin-1 with the outcome of patients was also described in other types of tumors, i.e. lung, breast and ovarian carcinoma [20, 21, 33, 34].

Regarding cofilin-1 intracellular localization, we demonstrated not only the increase of total levels of this protein, but also its higher expression in the nucleus of metastatic melanoma cells and MM with BI>2. Thus, besides the well-known function of cofilin-1 in migration when it is located at cytoplasm, we propose that cofilin-1 would be controlling other malignant features at the nucleus in melanoma. This could be mediated by actin efficient shuttling into the nucleus through its interaction with cofilin-1’s NLS [24]. In addition, cofilin-1 is part of the RNA polymerase II transcriptional machinery with a role in transcriptional elongation [35]. Consistent with our results, recent evidence showed an association between the nuclear localization of cofilin-1 and bladder cancer progression [36]. Furthermore, a partial nuclear translocation of phosphorylated/inactive cofilin-1 was observed in colon adenocarcinoma cell lines characterized by an invasive phenotype [37].

In conclusion, our results are an important step toward the validation process of cofilin-1 as a prognostic biomarker in melanoma. We suggest that cofilin-1 could be included in a molecular profile signature for melanoma prognosis based on genes or proteins involved in control of invasion and metastasis. Thus, further in-depth investigations related to cofilin-1 pathway in melanoma would be relevant to define potential biomarker signatures for earlier prognosis and development of new targeted therapies.

## MATERIALS AND METHODS

### Patient cohort and clinicopathological diagnosis

Formalin-fixed paraffin-embedded benign and malignant melanocytic lesions from patients diagnosed between 2000 and 2008 were obtained from the Pathological Anatomy Service, Hospital Italiano de Buenos Aires, Argentina (HIBA). The cohort consisted of 42 patients. The average age of the patients was 62.5 ± 20 years. Regarding sex, 52.4% of the patients were men and 42.8% were women. No significant differences in age and percentage of sex were found between groups of patients with the different diagnoses. The pathological diagnoses were reviewed and classified by two independent pathologists, according to World Health Organization criteria. Information such as histological type, melanoma stage, Breslow index (BI) and patient outcome were collected. Inclusion criteria were nevus, melanoma *in situ* (MIS), primary and metastatic melanoma previously diagnosed and with clinical follow-up of at least 5 years available. Besides, mitotic index was quantified as number of mitosis in 10 high power (400X magnification) fields as a measure of the growth rate.

This work has been carried out in accordance with The Code of Ethics of the World Medical Association (Declaration of Helsinki) for experiments involving human samples. The research program, including studies on archival and stored materials, was approved by the Research Protocols Ethics Committee of the HIBA (#1922).

The STROBE (Strengthening the Reporting of Observational Studies in Epidemiology) guidelines were used to ensure the reporting of this observational study (see Supplementary Information) [38, 39].

### Detection of cofilin-1 by immunohistochemistry

Tissue sections were deparaffinized with xylene and rehydrated through a series of graded alcohols. To detect cofilin-1, antigen retrieval was performed by incubating with proteinase K 40 μg/mL in TE buffer (Tris 50 mM, EDTA 1 mM and Triton X100 0.5%, pH 8) for 1 h at 37°C. Endogenous peroxidases were blocked with 5% hydrogen peroxide in methanol and to avoid nonspecific background staining, slides were incubated for 1 h with 1% bovine serum albumin (BSA) (Sigma) in phosphate-buffer saline (PBS). Rabbit polyclonal anti-cofilin-1 antibody 1:100 (Abcam, ab42824) was used. The immunogen sequence used by Abcam for obtaining ab42824 anti-cofilin antibody is a 14 amino acid peptide at the C-terminus of the human cofilin-1 protein. Samples were incubated with this antibody diluted 1:100, overnight at 4°C. After incubation, Super Sensitive IHC Detection Systems (BioGenex) kit, based on horseradish peroxidase-labeled polymer conjugated, was used following manufacturer’s instructions. After staining with diaminobenzidine solution, sections were dehydrated with alcohol, cleared in xylene and mounted (Entellan, Merck). In all cases, negative controls were obtained omitting the primary antibody, representing the background staining value in optical density (O.D.) measurements. Tissue sections obtained from normal skin and from breast cancer were used as negative and positive control tissues [17, 21], respectively.

The intensity of cofilin-1 IHC reaction was quantitatively measured in images obtained with an Olympus BX51 microscope coupled to a CCD camera (Olympus DP70). Five images were captured for each case on the same day by a single observer. Images were quantified as previously described [21], using Image J software (National Institutes of Health, Bethesda, USA) [40]. Results were expressed as the O.D. mean values in arbitrary units (A.U.).

### TCGA melanoma data set analysis

Data from 479 melanoma samples of a cohort of 471 patients generated by the TCGA Research Network (http://cancergenome.nih.gov/) [41, 42] was used for supplementary analysis of cofilin-1 at mRNA level (CFL1). Information regarding melanoma samples and CFL1, such as BI, overall patient survival status and mRNA expression z-scores (RNA Seq V2 RSEM), was obtained. Data about MM BI were clustered in MM BI<2 and MM BI>2.

### Survival data analysis

Standard Kaplan-Meier survival curves analyses were performed.

The survival curves were compared using the log-rank and Gehan-Breslow-Wilcoxon tests, and patients were clustered according to either the type of melanocytic lesions or cofilin-1 IHC expression level. Clustering of benign and malignant lesions include: nevi, MIS, MM BI<2 and MM BI>2. Regarding cofilin-1 immunocontent a cutoff of 0.08 A.U. of O.D. mean values was considered to cluster IHC samples between low and high cofilin-1 levels. This value was selected taking into account that the higher level of cofilin-1 expression of benign lesions was lower than 0.08. For TCGA data set, survival curves were performed in the same way, but CFL1 was divided in up-regulated or no altered according with a z-score threshold ± 2 and the time of the overall patient survival status was presented in months.

### Detection and subcellular localization of cofilin-1 by immunofluorescence

The intracellular localization of cofilin-1 was evaluated in a cellular model of melanoma developed in our laboratory [22] and in tissue sections of benign and malignant melanocytic lesions from patients. Immunocytofluorescence assay of cofilin-1 in non-metastatic A375 and metastatic A375-G10 cells was performed as previously described [22]. For immunohistofluorescence in patient samples, tissue sections were deparaffinized and rehydrated as described above. Microwave antigen retrieval was performed by placing the slides in 10 mM citrate buffer (pH 6.0) for 10 min. Tissue sections were blocked with 1% BSA in PBS and incubated overnight at 4°C with a rabbit polyclonal anti-cofilin-1 antibody 1:100 (Abcam, ab42824), then washed with PBS pH 8.2 and incubated with secondary fluorescein isothiocyanate (FITC)-conjugated anti-rabbit IgG (Sigma) for 1 h in the dark at room temperature. In order to evaluate cofilin-1 staining only in melanocytic lineage cells in the tissue sections of patients, after a second block with 1% BSA in PBS, samples were incubated overnight at 4°C with a mouse monoclonal HMB-45 antibody 1:400 (Cell Marque), then the samples were washed with PBS pH 8.2 and incubated with secondary rhodamine-conjugated anti-mouse IgG (Sigma) for 1 h in the dark at room temperature. Samples were washed, counterstained and mounted with 1 μg/mL 4’,6-diamidine-2’-phenylindole (DAPI, Sigma) and PBS/glycerol (80:20) in the dark. Cells were examined in an Olympus BX51 epifluorescence microscope utilizing immersion oil with a 100 (UPlanApo 100 X/1.35 oil) objective lens. For each sample of tissue sections from patients, FITC, rhodamine and DAPI images were serially captured by a CCD camera (Olympus DP70) at least five images were captured for each case on the same day by a single observer. FITC and DAPI images were captured in the same way for samples of melanoma cell model. In all cases negative controls were obtained omitting the primary antibody. A code number was given to each image. Random sampling methods were used to select the images to evaluate mean fluorescence of cofilin-1 in cytoplasms and nuclei. The original DAPI and cofilin-1 color images were merged for the selection of nuclei and cytoplasms. All cells from each selected images from non-metastatic A375 and metastatic A375-G10 cells and all the positive cells for melanocytic lineage staining in each selected image from tissue sections of patients were measured. To quantify mean fluorescence intensity for cofilin-1, Image J software (National Institutes of Health, Bethesda, USA) [40] was used. All images were converted to 8-bit grayscale, the background level was subtracted and the staining intensities were determined on the 0–255 greyscale. Random areas of equal size in each cytoplasm and nucleus were delimited in order to obtain the mean grey value, which represents mean fluorescence. An average of 1000 cells was evaluated per sample type. The relative nuclear/cytoplasmic value of mean fluorescence was calculated for each cell, both in the cell model of melanoma and in tissue sections from patients.

### Statistical analysis

Data are presented as mean ± SD. Significant changes were assessed using one-way analysis of variance and nonparametric Kruskal-Wallis test followed by Tukey’s or Dunn’s multiple comparison tests, respectively, to determine significant differences between group means. For cell experiments analysis, nonparametric *t* test was performed. P-values of less than 0.05 were considered significant for all tests. Regression analysis was performed when appropriate.

## SUPPLEMENTARY MATERIALS





## Abbreviations

AJCC: American Joint Commission on Cancer
A.U.: arbitrary units
BI: Breslow Index
BSA: bovine serum albumin
DAPI: 4’,6-diamidine-2’-phenylindole
FITC: fluorescein isothiocyanate
HIBA: Hospital Italiano de Buenos Aires
IHC: immunohystochemistry
MIS: melanoma *in situ*
MM: malignant melanoma
NLS: nuclear localization signal
O.D.: optical density
PBS: phosphate-buffer saline
STROBE: Strengthening the Reporting of Observational Studies in Epidemiology
TCGA: The Cancer Genome Atlas
TE: Tris-EDTA
TNM: tumor, lymph nodes and metastasis

## References

[1] American Cancer Society https://www.cancer.org/content/dam/cancer-org/research/cancer-facts-and-statistics/annual-cancer-facts-and-figures/2018/cancer-facts-and-figures-2018.pdf.

[2] Balch CM, Gershenwald JE, Soong SJ, Thompson JF, Atkins MB, Byrd DR, Buzaid AC, Cochran AJ, Coit DG, Ding S, Eggermont AM, Flaherty KT, Gimotty PA (2009). Final version of 2009 AJCC melanoma staging and classification. J Clin Oncol.

[3] Larkin J, Chiarion-Sileni V, Gonzalez R, Grob JJ, Cowey CL, Lao CD, Schadendorf D, Dummer R, Smylie M, Rutkowski P, Ferrucci PF, Hill A, Wagstaff J (2015). Combined nivolumab and ipilimumab or monotherapy in untreated melanoma. N Engl J Med.

[4] Chapman PB, Hauschild A, Robert C, Haanen JB, Ascierto P, Larkin J, Dummer R, Garbe C, Testori A, Maio M, Hogg D, Lorigan P, Lebbe C (2011). Improved survival with vemurafenib in melanoma with BRAF V600E mutation. N Engl J Med.

[5] Schreuer M, Jansen Y, Planken S, Chevolet I, Seremet T, Kruse V, Neyns B (2017). Combination of dabrafenib plus trametinib for BRAF and MEK inhibitor pretreated patients with advanced BRAFV600-mutant melanoma: an open-label, single arm, dual-centre, phase 2 clinical trial. Lancet Oncol.

[6] Ackerman A, Klein O, McDermott DF, Wang W, Ibrahim N, Lawrence DP, Gunturi A, Flaherty KT, Hodi FS, Kefford R, Menzies AM, Atkins MB, Long GV (2014). Outcomes of patients with metastatic melanoma treated with immunotherapy prior to or after BRAF inhibitors. Cancer.

[7] Sandru A, Voinea S, Panaitescu E, Blidaru A (2014). Survival rates of patients with metastatic malignant melanoma. J Med Life.

[8] Mandala M, Massi D (2014). Tissue prognostic biomarkers in primary cutaneous melanoma. Virchows Arch.

[9] Garbe C, Peris K, Hauschild A, Saiag P, Middleton M, Bastholt L, Grob JJ, Malvehy J, Newton-Bishop J, Stratigos AJ, Pehamberger H, Eggermont AM (2016). Diagnosis and treatment of melanoma. european consensus-based interdisciplinary guideline - update 2016. Eur J Cancer.

[10] Ohsie SJ, Sarantopoulos GP, Cochran AJ, Binder SW (2008). Immunohistochemical characteristics of melanoma. J Cutan Pathol.

[11] Pollock PM, Harper UL, Hansen KS, Yudt LM, Stark M, Robbins CM, Moses TY, Hostetter G, Wagner U, Kakareka J, Salem G, Pohida T, Heenan P (2003). High frequency of BRAF mutations in nevi. Nat Genet.

[12] Kashani-Sabet M (2014). Molecular markers in melanoma. Br J Dermatol.

[13] Sidransky D (2002). Emerging molecular markers of cancer. Nat Rev Cancer.

[14] DePeralta DK, Boland GM (2015). Melanoma: Advances in Targeted Therapy and Molecular Markers. Ann Surg Oncol.

[15] Bravo-Cordero JJ, Hodgson L, Condeelis J (2012). Directed cell invasion and migration during metastasis. Curr Opin Cell Biol.

[16] Sidani M, Wessels D, Mouneimne G, Ghosh M, Goswami S, Sarmiento C, Wang W, Kuhl S, El-Sibai M, Backer JM, Eddy R, Soll D, Condeelis J (2007). Cofilin determines the migration behavior and turning frequency of metastatic cancer cells. J Cell Biol.

[17] Wang W, Eddy R, Condeelis J (2007). The cofilin pathway in breast cancer invasion and metastasis. Nat Rev Cancer.

[18] Martin SK, Kamelgarn M, Kyprianou N (2014). Cytoskeleton targeting value in prostate cancer treatment. Am J Clin Exp Urol.

[19] Popow-Wozniak A, Mazur AJ, Mannherz HG, Malicka-Blaszkiewicz M, Nowak D (2012). Cofilin overexpression affects actin cytoskeleton organization and migration of human colon adenocarcinoma cells. Histochem Cell Biol.

[20] Castro MA, Dal-Pizzol F, Zdanov S, Soares M, Muller CB, Lopes FM, Zanotto-Filho A, da Cruz Fernandes M, Moreira JC, Shacter E, Klamt F (2010). CFL1 expression levels as a prognostic and drug resistance marker in nonsmall cell lung cancer. Cancer.

[21] Muller CB, de Barros RL, Castro MA, Lopes FM, Meurer RT, Roehe A, Mazzini G, Ulbrich-Kulczynski JM, Dal-Pizzol F, Fernandes MC, Moreira JC, Xavier LL, Klamt F (2011). Validation of cofilin-1 as a biomarker in non-small cell lung cancer: application of quantitative method in a retrospective cohort. J Cancer Res Clin Oncol.

[22] Bracalente C, Salguero N, Notcovich C, Müller CB, Da Motta LL, Klamt F, Ibañez IL, Durán H (2016). Reprogramming human A375 amelanotic melanoma cells by catalase overexpression: reversion or promotion of malignancy by inducing melanogenesis or metastasis. Oncotarget.

[23] Kanellos G, Frame MC (2016). Cellular functions of the ADF/cofilin family at a glance. J Cell Sci.

[24] Munsie LN, Desmond CR, Truant R (2012). Cofilin nuclear-cytoplasmic shuttling affects cofilin-actin rod formation during stress. J Cell Sci.

[25] Miyamoto K, Gurdon JB (2013). Transcriptional regulation and nuclear reprogramming: roles of nuclear actin and actin-binding proteins. Cell Mol Life Sci.

[26] Bravo-Cordero JJ, Magalhaes MA, Eddy RJ, Hodgson L, Condeelis J (2013). Functions of cofilin in cell locomotion and invasion. Nat Rev Mol Cell Biol.

[27] Boland GM, Gershenwald JE (2016). Principles of melanoma staging. Cancer Treat Res.

[28] Kirkwood JM, Moschos S, Wang W (2006). Strategies for the development of more effective adjuvant therapy of melanoma: Current and future explorations of antibodies, cytokines, vaccines, and combinations. Clin Cancer Res.

[29] Rowe CJ, Khosrotehrani K (2016). Clinical and biological determinants of melanoma progression: Should all be considered for clinical management?. Australas J Dermatol.

[30] Chen X, Guo W, Xu XJ, Su F, Wang Y, Zhang Y, Wang Q, Zhu L (2017). Melanoma long non-coding RNA signature predicts prognostic survival and directs clinical risk-specific treatments. J Dermatol Sci.

[31] Caputo E, Maiorana L, Vasta V, Pezzino FM, Sunkara S, Wynne K, Elia G, Marincola FM, McCubrey JA, Libra M, Travali S, Kane M (2011). Characterization of human melanoma cell lines and melanocytes by proteome analysis. Cell Cycle.

[32] Guan M, Chen X, Ma Y, Tang L, Guan L, Ren X, Yu B, Zhang W, Su B (2015). MDA-9 and GRP78 as potential diagnostic biomarkers for early detection of melanoma metastasis. Tumour Biol.

[33] Shaheed SU, Rustogi N, Scally A, Wilson J, Thygesen H, Loizidou MA, Hadjisavvas A, Hanby A, Speirs V, Loadman P, Linforth R, Kyriacou K, Sutton CW (2013). Identification of stage-specific breast markers using quantitative proteomics. J Proteome Res.

[34] Nishimura S, Tsuda H, Kataoka F, Arao T, Nomura H, Chiyoda T, Susumu N, Nishio K, Aoki D (2011). Overexpression of cofilin 1 can predict progression-free survival in patients with epithelial ovarian cancer receiving standard therapy. Hum Pathol.

[35] Obrdlik A, Percipalle P (2011). The F-actin severing protein cofilin-1 is required for RNA polymerase II transcription elongation. Nucleus.

[36] Hensley PJ, Zetter D, Horbinski CM, Strup SE, Kyprianou N (2016). Association of epithelial-mesenchymal transition and nuclear cofilin with advanced urothelial cancer. Hum Pathol.

[37] Ferraro A, Boni T, Pintzas A (2014). EZH2 regulates cofilin activity and colon cancer cell migration by targeting ITGA2 gene. PLoS One.

[38] Vandenbroucke JP, von Elm E, Altman DG, Gotzsche PC, Mulrow CD, Pocock SJ, Poole C, Schlesselman JJ, Egger M (2007). Strengthening the reporting of observational studies in epidemiology (STROBE): explanation and elaboration. PLoS Med.

[39] von Elm E, Altman DG, Egger M, Pocock SJ, Gotzsche PC, Vandenbroucke JP (2007). The strengthening the reporting of observational studies in epidemiology (STROBE) statement: Guidelines for reporting observational studies. PLoS Med.

[40] Rasband WS https://imagej.nih.gov/ij/.1997-2016.

[41] Gao J, Aksoy BA, Dogrusoz U, Dresdner G, Gross B, Sumer SO, Sun Y, Jacobsen A, Sinha R, Larsson E, Cerami E, Sander C, Schultz N (2013). Integrative analysis of complex cancer genomics and clinical profiles using the cBioPortal. Sci Signal.

[42] Cerami E, Gao J, Dogrusoz U, Gross BE, Sumer SO, Aksoy BA, Jacobsen A, Byrne CJ, Heuer ML, Larsson E, Antipin Y, Reva B, Goldberg AP (2012). The cBio cancer genomics portal: an open platform for exploring multidimensional cancer genomics data. Cancer Discov.

