# Supplementary material for: Cofilin-1 levels and intracellular localization are associated with melanoma prognosis in a cohort of patients
Source: Oncotarget. 2018 May 8;9(35):24097–108. doi: 10.18632/oncotarget.25303 (PMC5963619; doi:10.18632/oncotarget.25303)
Supplement: Supplementary file 1 [file oncotarget-09-24097-s001.pdf]

Cofilin-1 levels and intracellular localization are associated with melanoma prognosis in a cohort of patients

SUPPLEMENTARY MATERIALS

Supplementary Information: Report of the cohort observational study of melanocytic lesions according to STROBE Statement[1, 2].

See Supplementary File 1
