# Supplementary material for: Cofilin-1 levels and intracellular localization are associated with melanoma prognosis in a cohort of patients
Source: Oncotarget. 2018 May 8;9(35):24097–108. doi: 10.18632/oncotarget.25303 (PMC5963619; doi:10.18632/oncotarget.25303)
Supplement: Supplementary file 2 [file oncotarget-09-24097-s002.docx]

Supplementary Information:

Report of the cohort observational study of melanocytic lesions according to STROBE Statement ^1, 2^

|  |  | **Item** | | | |  | |  |  | |
| --- | --- | --- | --- | --- | --- | --- | --- | --- | --- | --- |
|  |  | **No** |  | | |  |  | | |  |
|  | **Title and abstract** | 1 | (*a*) Cofilin-1 levels and intracellular localization are associated with prognosis of melanoma: an observational study | | |  |  |  |  |  |
|  |  |  | (*b*) Background: Melanoma is an aggressive cancer with highly metastatic ability. We propose cofilin-1, a key protein in the regulation of actin dynamics and migration, as a prognostic marker.  Methods: We determined cofilin-1 levels in a retrospective cohort of patients with melanomas and benign lesions of melanocytes (nevi) by immunohistochemistry (IHC). Kaplan-Maier survival curves were performed, clustering patients according to either the type of melanocytic lesions and cofilin-1 IHC level. A melanoma cohort from TCGA Research Network was analysed, comparing samples with up-regulated vs non altered CFL1 mRNA expression. As cofilin-1 has a dual function depending on its intracellular localization, we evaluated nuclear and cytoplasmic levels of cofilin-1 in melanoma and nevi samples by immunofluorescence.  Results: Higher cofilin-1 levels were found in malignant melanoma (MM) with Breslow Index (BI)>2 vs MM with BI<2, melanoma in situ (MIS) and nevi and also in MM with metastasis vs MM without detected metastasis. Kaplan-Meier curves demonstrated worse prognosis of patients with high vs low cofilin-1 levels. Supporting these results, TCGA database analysis of melanoma also showed low survival in patients with upregulated cofilin-1 mRNA. Remarkably, an increase in nuclear/cytoplasmic cofilin-1 mean fluorescence ratio was observed in MM with BI>2 vs MM with BI<2, MIS and nevi, in relation with melanoma progression.  Conclusion: An association of cofilin-1 levels with malignant features and an inverse correlation with survival were demonstrated. Our study suggests that not only the higher levels of cofilin-1, but also its nuclear localization can be markers of worse outcome of patients with melanoma. | | |  |  |  |  |  |
|  | **Introduction** |  |  |  |  |  |  |  |  |  |
|  | Background/rationale | 2 | Melanoma is an aggressive cancer with highly metastatic ability. Conventional tissue biomarkers, such as BI, ulceration, mitotic rate and lymph node positivity, remain the backbone prognostic indicators in melanoma. However, since recurrence rate is largely independent from stages defined by morphological and morphometric criteria, there is a strong need for identification of additional robust prognostic factors to support decision-making processes. Proteins involved in transforming melanoma cells into a migratory phenotype can be proposed as prognostic markers. In this sense, high levels of cofilin-1, which favors migration by inducing cycles of actin polymerization/depolymerization, has been found in invasive lung and breast cancer. We previously reported high levels of cofilin-1 in a melanoma metastatic cell line.  In addition to its function in cell migration, cofilin-1 also has a nuclear localization signal, which allows its involvement in other functions at the nucleus. However, the specific biological function of nuclear translocation of cofilin-1 is still unclear in cancer progression.  These previous evidences lead us to propose an association between cofilin-1 and metastatic ability and worse outcome in melanoma. | | |  |  |  |  |  |
|  | Objectives | 3 | The objective of this study was to determine the levels and subcellular localization of cofilin-1 in melanoma human samples with different degrees of malignancy, in order to evaluate the hypothesis that this protein could be used as predictor and prognostic marker of advanced melanoma. | | |  |  |  |  |  |
|  | **Methods** |  |  |  |  |  |  |  |  |  |
|  | Study design | 4 | This is a comparative study of the levels and subcellular localization of cofilin-1 in patients with different stages of melanoma and benign melanocytic lesions. The association among cofilin-1 and histological type, melanoma stage, Breslow index, mitotic index, metastasis presence and patient outcome was evaluated. | | |  |  |  |  |  |
|  | Setting | 5 | This study was performed with archival formalin-fixed paraffin-embedded tissue sections of benign and malignant melanocytic lesions from patients diagnosed between 2000 and 2008 with clinical follow-up of at least 5 years available. These samples were obtained from the Pathological Anatomy Service, Hospital Italiano de Buenos Aires, Argentina (HIBA). | | |  |  |  |  |  |
|  | Participants | 6 | (*a*) Inclusion criteria were nevus, melanoma in situ (MIS), primary and metastatic melanoma previously diagnosed and with clinical follow-up of at least 5 years available. Regarding sex and age, the inclusion criteria considered a balanced percentage between groups of patients with the different diagnoses. The pathological diagnoses were reviewed and classified by two independent pathologists, according to World Health Organization criteria. | | |  |  |  |  |  |
|  |  |  | (*b*) Matching criteria for analyzing cofilin-1 levels considered the comparison between melanomas and benign melanocytic lesions; melanomas with Breslow index (BI) < 2 vs melanomas with BI > 2; with and without metastasis and among different stages and types of melanoma. | | |  |  |  |  |  |
|  |  |  |  | | |  |  |  |  |  |
|  | Variables | 7 | The following parameters of samples were taken into account to compare cofilin-1 levels: histopathological diagnosis, which included: benign vs malignant melanocytic lesions; melanoma stage (TNM); histological type (nevus, in situ melanoma and different types of malignant melanoma), Breslow Index, number of mitosis per field; presence of metastasis and survival at 5 years. | | |  |  |  |  |  |
|  |  |  |  | | |  |  |  |  |  |
|  | Data sources/ | 8 | All the histopathological data were provided by the Pathological Anatomy Service, Hospital Italiano de Buenos Aires (HIBA) and confirmed by the pathologist who participated in this study. Thus, these data were reviewed and classified by two independent pathologists, according to World Health Organization criteria. Regarding clinical follow up, presence of metastasis and survival, these information were provided by the Dermatology Service, HIBA. | | |  |  |  |  |  |
|  | Bias | 9 | To avoid some potential sources of bias, we considered in the study design that there were not significant differences among groups in the proportion of sex and ages of patients. | | |  |  |  |  |  |
|  | Study size | 10 | The study size was limited by the availability of samples from patients with the complete information we needed for the study. It was compensated by studying cofilin-1 expression in samples of melanoma from TCGA database. | | |  |  |  |  |  |
|  | Quantitative variables | 11 | Regarding malignant melanomas (MM), they were grouped in MM with BI < 2 and MM with BI > 2. This criterion was chosen considering the likelihood of invasion and metastasis induction. Regarding cofilin-1 levels, a cutoff of 0.08 A.U. of O.D. mean values was considered to cluster IHC samples between low and high cofilin-1. This value was selected taking into account that the higher level of cofilin-1 expression of benign lesions was lower than 0.08. | | |  |  |  |  |  |
|  | Statistical methods | 12 | Normality tests and descriptive statistics (considering minimum, maximum, quartiles, mean, SD, Skewness, Kurtosis and 95% CI) were performed before any statistical comparison analysis among groups. When data passed normality tests, significant changes were assessed using one-way analysis of variance followed by Tukey’s multiple comparison tests to determine significant differences between group means. On the other hand, when data could not pass normality tests, nonparametric Kruskal-Wallis test followed by Dunn’s multiple comparison tests were performed. P-values of less than 0.05 were considered significant for all tests.  Data about MM BI were clustered in MM BI<2 and MM BI>2 for both our cohort of samples and the TCGA data set. Clustering of benign and malignant lesions included: nevi, MIS, MM BI<2 and MM BI>2. Data about mitosis/field were clustered in <1, 1-5 and >5 and data about metastasis in presence or absence. The AJCC TNM system was use to classified tumor stages. Regarding cofilin-1 immunocontent, as already mentioned a cutoff of 0.08 A.U. of O.D. mean values was considered to cluster IHC samples between low and high cofilin-1 levels.  Standard Kaplan-Meier survival curves analyses were performed. The survival curves were compared using the log-rank and Gehan-Breslow-Wilcoxon tests, and patients were clustered according to either the type of melanocytic lesions and cofilin-1 IHC expression level. For TCGA data set, survival curves were performed in the same way, but CFL1 was divided in up-regulated or no altered according with a z-score threshold ± 2 and the time of the overall patient survival status was presented in months.  Regression analysis were done using Spearman's Rho statistic in order to evaluate the strength and direction of the relationship between survival percentage and cofilin-1 immunocontent. | | |  |  |  |  |  |
|  |  |  |  |  |  |  |  |  |  |  |
|  | **Results** |  |  |  |  |  |  |  |  |  |
|  | Participants | 13 | (a) The cohort used for this study consisted in 42 patients. All of them were eligible according to the inclusion criteria. | | |  |  |  |  |  |
|  |  |  | (b) All of the samples were used in the study. | | |  |  |  |  |  |
|  | Descriptive data | 14 | (a) All of the samples come from patients treated at the HIBA, we do not have demographic and social information. The average age of the patients was 62.5 ± 20 years. Regarding sex, 52.4% of the patients were men and 42.8% were women. No significant differences in age and percentage of sex were found between groups of patients with the different diagnoses. The amount of patients with the different tumor stages and histological types are detailed in Table 1 of the manuscript. Regarding BI, 40% of the malignant melanomas presented BI<2 and 60% BI>2 33.3% of melanomas presented metastasis and 51,5% did not present detectable metastasis. | | |  |  |  |  |  |
|  |  |  | (b) The number of patients with missing data for sex, histological type and tumor stage are indicated in Table 1. For presence of metastasis, 5 patients were undetermined. There were no missing data for BI of malignant melanoma and for survival at 5 years. | | |  |  |  |  |  |
|  |  |  | (c) The follow up was 5 years for all patients. | | |  |  |  |  |  |
|  | Outcome data | 15 | A summary of the outcome data in percentage of low (<0.08) and high (>0.08) cofilin-1 immunocontent for each clustering evaluated is presented in Table 1.  Outcome event evaluated was survival for 5 years. These outcome data are represented in Figure 2b. Survival data at 5 years showed 93.75% of survival for patients with low cofilin-1 and 23.49% of survival for patients with high levels of cofilin-1. | | |  |  |  |  |  |
|  | Main results | 16 | (*a*) A significant association between high levels of cofilin-1 and MM advanced stages (p < 0.001), metastasis (p < 0.01), increased number of mitosis per field (p < 0.05) and BI (p < 0.05) was found.  Patients with high cofilin-1 levels presented lower survival rate at 5 years than patients with low cofilin-1 levels (p<0.0001). A significant negative correlation between survival percentage and cofilin-1 immunocontent (R=-0.73, p=0) was found.  Regarding intracellular localization, cofilin-1 was found not only at cytoplasm, but also at the nucleus of melanomas and only at cytoplasm in benign lesions of melanocytes. Nuclear/cytoplasm ratio was significant higher in MM BI>2 compared with MM BI<2 and MIS. | | |  |  |  |  |  |

(*b*) The levels of cofilin-1 were categorized as low when the immunocontent in A.U. was <0.08 and high when it was <0.08. This boundary of 0.08 was defined from the levels of cofilin-1 of benign lesions.

| Other analyses | 17 | A supplementary analysis of cofilin-1 expression at mRNA level was performed using a TCGA melanoma dataset (a cohort of 471 patients). Information regarding melanoma samples and CFL1, such as BI, overall patient survival status and mRNA expression z-scores (RNA Seq V2 RSEM), was considered from this database. | |
| --- | --- | --- | --- |
|  |  |  | |
| **Discussion** |  |  | |
| Key results | 18 | | Cofilin-1 levels were found associated with malignancy in a cohort of melanoma patients, with an inverse correlation between cofilin-1 levels and survival. Moreover, TCGA data base analysis showed that melanomas with up-regulated cofilin-1 mRNA presented worse outcome.  Regarding intracellular localization, we found that melanomas and metastatic cells not only presented cytoplasmic cofilin-1, but also showed this protein at the nucleus with higher nuclear/cytoplasm ratio of cofilin-1 for more malignant melanoma. |
| Limitations | 19 | One limitation of this study was the availability of patient samples with the required information and clinical follow-up. Although the limited study size, statistically significant results were obtained. Besides, in order to surpass this sample size limitation, TCGA database analysis of cofilin-1 expression in melanoma was performed. | |
| Interpretation | 20 | Our results suggest cofilin-1 as a prognostic marker of melanoma outcome. We also suggest that cofilin-1 could have another role in malignant progression, besides its involvement in migration, due to the finding of high levels of cofilin-1 at the nucleus. However, considering the limitation of the study size, these results should be validated with a large cohort study.  Other studies in different types of cancer are in agreement with the involvement of cofilin-1 in malignant progression and worse outcome. | |
| Generalisability | 21 | Our results are the first step to propose cofilin-1 as a prognostic biomarker in melanoma. To validate the use of this marker in melanoma, the ideal design should be to conduct a large prospective cohort study. | |
| **Other information** |  |  | |
| Funding | 22 | This work was supported by National Agency of Scientific and Technological Promotion, Argentina (International Cooperation PICT-CABBIO 2014-0818 and PICT 2014-1557); MCTI/CNPQ/CBAB International Cooperation in Biotechnology, Brazil (465113/2014-1); National Atomic Energy Commission, Argentina; Florencio Fiorini Foundation, Argentina. | |

References

1. von Elm E, Altman DG, Egger M, Pocock SJ, Gotzsche PC, Vandenbroucke JP. The Strengthening the Reporting of Observational Studies in Epidemiology (STROBE) statement: guidelines for reporting observational studies. PLoS Med 2007;4(10): e296.

2. Vandenbroucke JP, von Elm E, Altman DG, et al. Strengthening the Reporting of Observational Studies in Epidemiology (STROBE): explanation and elaboration. PLoS Med 2007;4(10): e297.
